# The role of HRECs in regulating medical research: from peer review to regulation

**DOI:** 10.1007/s40592-025-00248-z

**Published:** 2025-05-15

**Authors:** Lisa Eckstein, Jenny C. Kaldor, Cameron Stewart

**Affiliations:** 1 College of Arts Law and Education, University of Tasmania, Hobart, Australia; 2 Bellberry Ltd, Eastwood, Australia; 3 Department of Health, Hobart, TAS Australia; 4 Sydney Law School, University of Sydney, Darlington, Australia

**Keywords:** Clinical trials, Decision making, Ethics committees, Research, Peer review, Waivers of consent

## Abstract

In Australia, Human Research Ethics Committees (HRECs) play a ubiquitous role reviewing human subjects research, as do Institutional Review Boards in the US and elsewhere. While HRECs were established as peer review bodies, we argue they should now be characterised a ‘devolved regulator’ within the broader context of the regulatory state. We evidence HRECs’ regulatory role through three examples of current responsibilities. By categorising HRECs as a regulator, we are able to assess their role through a regulatory lens. Drawing on Reeve and Magnusson’s ‘regulatory scaffolding’ approach, we suggest key ways in which the role provided by HRECs could be improved. These include setting clear roles and responsibilities HREC review; ensuring HREC accountability for the substantive aspects of their decision making; and accountability for trial sponsors who seek review of trials under the Clinical Trials Notification Scheme. Deficits in the above must incur a credible expectation of escalation and review.

## Introduction

For nearly five decades, human research ethics committees (“HRECs”) (in the US, referred to as institutional review boards (“IRBs”)) have been seen as a necessary component of the modern research enterprise. Introduced in the American research environment in the 1960s, HRECs spread quickly into Europe and the Pacific, and later into Africa, Asia and the Middle-East (Guillemin et al. 2012). HRECs are now a ubiquitous part of research involving human and non-human animals, and their operation has been itself researched, particularly in relation to their structure and function, their internal processes and the way they make decisions (McNeill et al. 1990; Hayes et al. 1995; Guillemin et al. 2012). But while this research oft repeats the notion that HRECs are a part of research regulation, that have not been scrutinised *as a form of regulation* – that is, through a regulatory lens.

This article bridges that gap by examining HRECs through the lens of regulatory scholarship. Regulatory scholarship is an interdisciplinary body of scholarship that examines the theory and practice of governance, with a particular focus on how different forms of rule-making, surveillance and enforcement can manage and promote economic and social activity (Levi-Faur et al. 2021). Regulation theory is an especially useful discipline for understanding the relationships between governments, decision-makers, and those affected by decisions. At an empirical level, regulatory scholars have demonstrated that different regulatory forms entail different costs, benefits, and burdens, for different regulatory actors (Losoncz 2017). At the normative level, they have sought to offer guidance to regulators about when and how to deploy each regulatory strategy (Reeve and Magnusson 2015).

The paper will detail the history—and increasing ubiquity—of HREC review for human subject research. We go on to characterise HRECs as “devolved regulators” within the regulatory state, using three examples of current HREC functions: clinical trial product safety reviews under the Australian Clinical Trial Notifications scheme; authorising waivers of consent for the release of personal information under federal, state and territory privacy acts; and authorising projects where the participants lack legal capacity to consent to the research. We conclude by suggesting reforms to HREC operations in light of its regulatory nature, drawing, in particular, on the “regulatory scaffolding” approach developed by Reeve and Magnusson. Although the discussion and proposed reforms are targeted to the Australian context, they likely have relevance to research ethics committees more broadly.

## Background

Independent review of the ethical acceptability of research first started in the United States through 1963 guidelines issued by the United States National Institutes of Health, which required independent committees to assess the scientific merit of research proposals and, eventually, wider social considerations (Stark 2012). These were the precursors to Institutional Review Boards (IRBs), review by which is now mandatory for most human subjects research conducted by US institutions. Compliance is required either through the Code of Federal Regulations, Title 45, Part 46 (45 CFR 46, “the Common Rule”) for federally funded research or—for privately sponsored research—21 CFR 50, 56.

In Australia, “peer group assessment” of human research was first recommended in 1972 and became mandatory in 1976 for all applications seeking research funding through the federal National Health and Medical Research Council (NHMRC) (Chalmers 2001). Since this time, eligibility for research funding has acted as a key incentive for institutions to establish research ethics committees, now termed Human Research Ethics Committees (HRECs) (Dodds 2000). Compliance is required either through funding agreements between research institutions and the NHMRC or through therapeutic goods laws requiring HREC approval for all research involving unapproved therapeutic goods. HREC approval is also required for certain uses of personal information without consent and, in some states and territories, for research involving persons without decision-making capacity.

The trajectory for IRBs in the US and HRECs in Australia has been one of increasing decision-making specificity and complexity. Professor Sarah Babb has chronicled the transformation of US IRBs from “peer review committees” guided by informal norms to committees charged with “more extensive regulatory and administrative responsibilities” (Babb 2020, 20). She notes that

With the transformation of internal policies into regulations, and with each subsequent update, these mandated procedures increased in volume and complexity. The 1981 regulations were over 10,000 words long, more than double the length of the 1974 version; the 1991 Common Rule… weighed in at more than 12,000 words (Babb 2020, 20).

The result of these changing and growing set of rules has been “an intricate, overlapping set of decision criteria, sub-criteria, and exceptions that could mystify even the most motivated faculty board member” (Babb 2020, 20).

Australian HRECs have undergone a similar transformation. Initial requirements for committee operation and principles for review, set out in the 1966 NHMRC *Statement on Human Experimentation*, were somewhat minimal. The only written stipulation in relation to the composition of these committees, for example, was the need to appoint one person not connected with the institution (Chalmers 2001). More detailed requirements for ethics committee composition and functions was issued with the revised *Statement on Human Experimentation* in 1982. A new Supplementary Note specified the need for Committees to comprise men and women, and to include a person not associated with the institution, a minister of religion, a lawyer, a medical graduate with research experience, and a male and female lay person (National Health and Medical Research Council 1982, Supplementary Note 1). The Supplementary Note advised that Committees should “establish and maintain communication with the national Medical Research Ethics Committee [a then advisory committee to the NHMRC] and provide access, upon request, to information in the institutional ethics committee”s register” (National Health and Medical Research Council 1982, Supplementary Note 1, 1(d)). The Statement underwent further review in 1992, now consisting of a brief introductory paragraph, as well as 13 short statements of ethical principles, and seven supplementary notes of between one and five pages (Dodds 2000).

Since 1999, principles for HREC review have been set out in the *National Statement on Ethical Conduct in Research Involving Humans* (1999, updated 2018). The National Statement has been described as shifting “from an “aspirational ethic” enunciating professional ideals to a greater emphasis on the practical regulation of the process of ethical review by research ethics committees” (Dodds 2000, 5). In order to provide this “regulatory code of ethical practice” (Dodds 2000, 10), much more detailed guidance was necessary. At the time of writing, the most recent version of the *National Statement* numbers 114 pages, including lengthy requirements for committee procedures and processes, committee composition, and record keeping (National Health and Medical Research Council et al. 2007, chap. 5.1, 5.2). It is worth recognising that some of this new guidance consists of substantive ethical guidance and increasing amounts of formalised procedure and compliance mechanisms.

Research ethics committees have been increasingly entrusted with highly specific and consequential decisions. In the US, since 2003, Institutional Review Boards (IRBs) have been required to approve waivers or alterations to the Health Insurance Portability and Accountability Act of 1996 (HIPAA) Privacy Rule for uses and disclosures of personal health information for research (Department of Health and Human Services 2003). IRBs also have been given extensive responsibilities when it comes to the review of waivers of the requirement to seek informed consent of participants for research conducted in emergency settings. Under 21 CFR § 50.24, an IRB must find and document additional protections of the rights and welfare of subjects, including the following:

- Participants are in a life-threatening situation, available treatments are unproven or unsatisfactory, and valid scientific evidence is necessary to determine the safety and effectiveness of particular interventions.
- It is not feasible to obtain informed consent.
- Participation in the study holds the prospect of direct benefits to participants and entails only reasonable risks.
- The study could not practicably be carried out without the waiver.
- The researcher will contact a legally authorized representative for each participant, if feasible, to seek consent, and will provide IRBs with information about their efforts to make such contact in continuing review.
- Informed consent will be used when feasible with participants or their legally authorized representatives, including opportunities for family members to object to participation.
- Researchers have undertaken consultation and public disclosure activities in the communities in which the research will be conducted.
- An independent data and safety monitoring board has been established to oversee the study.

IRB determinations and documentation must be available for review by the Food and Drug Administration for at least three years after the study has been completed.

In Australia, HREC decision-making responsibilities are arguably even greater. Perhaps the most compelling example is HREC responsibility for product safety assessments. Since the introduction of the Clinical Trial Notification (CTN) scheme in 1991, HRECs have been entrusted with assessing the safety of the vast majority of unapproved therapeutic goods being used in clinical trials. This HREC assessment supports the use of a *notification* rather than *assessment* scheme by the governmental product regulator: the Therapeutic Goods Administration (TGA). A related decision-making responsibility is the assessment of applications by medical practitioners to provide access to unapproved therapeutic goods under the TGA “Authorised Prescriber” scheme. Under this scheme, HRECs are responsible for providing an initial assessment of the suitability of the proposed use as well as the expertise of the applicant to provide such use (Therapeutic Goods Administration 2020).

Additional HREC responsibilities come through the federal and state privacy laws. Sections 16A and 95A of the *Privacy Act 1988* (Cth) permits the collection, use, or disclosure of personal health information without consent if “the use or disclosure is necessary for research, or the compilation or analysis of statistics, relevant to public health or public safety” and in accordance with guidelines issued by the National Health and Medical Research Council or a prescribed authority. Similar regulation also exists at the state level (Jeanneret et al. 2019). The Guidelines issued under this legislation require that an HREC must give approval for the collection, use or disclosure of health information for the purpose of the proposed research.

Some Australian state and territory laws also embed HREC review into requirements for consent by guardianship authorities for the inclusion of incapacitated adults and young people (over the age of 16) in research. For example, under s 81 of the Victorian *Medical Treatment, Planning and Decision Act 2016* (Vic), HREC approval is a necessary step in the process of having guardianship approval for projects that involve the inclusion incapacitated research participants in circumstances where the participants have no medical treatment decision-maker. Section 81 of this Act also requires HRECs to collect and maintain records of who has been enrolled into such research.

## Characterising HRECs as “devolved regulators”

### Background to the regulatory state

The context for this discussion is the *regulatory state*, a mode of governance that has characterised liberal democracies (Ford 2017, 52). During the 1970–80 s a number of legal theories of the regulatory state began to emerge (for e.g., Unger”s theory of the “Legal Order” (Unger 1977), Kamenka and Tay”s model of *Gemeinschaft*, *Gesellschaft* and administrative-bureaucratic trends within law (Kamenka and Erh-Soon Tay 1975), and Nonet and Selznick”s theory of the emergence of “responsive law” (Nonet and Selznick 1978, 17) that defined the role of governments as moving away from classical liberal economics based on formal laws of command and control and towards forms of bureaucratic-administrative order where the government’s primary role was to manage economic activity and risk. In the decades after the Second World War, that management was performed largely by the government as the “welfare state” but by the 1990s a new “regulatory state paradigm” had replaced the older welfare state model (Ford 2017, 52). In a regulatory state, the State is said to have “receded”: retaining a central directing role, but exercising control in different ways than previously; top-down “governing” giving way to a more decentralised and diffused “governance” (Moran 2002; Bovaird and Löffler 2003; Although NB Bartle and Vass 2007, 316: “there are few terms which are as vague in social science and in practice as “governance”). This has two important implications: first, the State is no longer the main or only regulatory actor; secondly, traditional rule-of-law-based command-and-control instruments – primarily, mandatory legislation backed by penalties – are no longer the main or only form of regulation.

For the purposes of this article, we adopt Black’s definition of “regulation, or regulatory governance”, as:

the organised attempt to manage risks or behaviour in order to achieve a publicly-stated objective or set of objectives; a regulatory system consists of the (sometimes shifting) set of interrelated actors who are engaged in such attempts and their interactions with one another and the dynamic institutional and organisational environment in which they sit (Black 2014, 4).

Characteristic phenomena of the regulatory state include outsourced public service delivery functions (Black 2014, 14); deregulated markets (Ostry et al. 2016); the State as a facilitator, “steering” rather than “rowing” the ship of government (Osborne and Gaebler 1992); and the proliferation both of new, “flexible” forms of regulation (Bartle and Vass 2007; Scott 2010), and—most importantly for our purposes—of new, devolved or decentralised *regulators* (Ford 2017, 62).

While new, devolved regulators will often use new, flexible forms of regulation, the two concepts are not interchangeable. Flexible regulation refers to the expanded range of strategies available to regulators seeking to foster compliance with policy objectives (Breger 1996). Sitting between the polar extremes of command-and-control (also known as “hard”) regulation, and no regulation at all, these strategies tend to be described as “soft” regulation (Freiberg 2010). As Freiberg notes (Freiberg 2010, 22–36), these strategies include

- self-regulation, in which the regulated industry formulates, implements, and enforces its own rules and codes of conduct (Black 2014, 118 Black refers to mandated self-regulation, sanctioned self-regulation, coerced self-regulation and voluntary self-regulation);
- quasi-regulation, in which government uses a range of instruments – including codes, rules, and guidelines – to exert pressure on industry to comply, but these instruments do not constitute legislative or executive regulations (Freiberg 2010, 186);
- co-regulation, in which the regulated industry formulates and implements its own rules, but the State provides legislative support for enforcement; and sometimes
- meta-regulation: the regulation of regulation, i.e., processes that allow for the auditing or supervision of other regulatory strategies (Freiberg 2010, 33).

These “soft” strategies are often associated with Ayres and Braithwaite’s influential concept of “responsive regulation” (as represented by the famous “regulatory pyramid”), which proposed that governments should start with the “softer” forms of regulation—at the base of the pyramid—and then gradually work their way up to “harder” regulation, as and when needed (Ayres and Braithwaite 1992). Importantly, the success of the softer forms of regulation is not independent of the harder ones: rather, their success depends on the genuine possibility of escalation to a regulatory form higher up the pyramid.

The concept of devolved regulators refers to bodies other than the traditional institutions of central government, which are nevertheless undertaking regulatory governance—recall Black’s definition above, “the organised [management of] risks or behaviour in order to achieve a publicly-stated objective or set of objectives”. In the public sector, this concept is often associated with “agencification” (McGauran et al. 2005), or with the contracting out (“outsourcing”) of services (Birch and Siemiatycki 2016). In either case, a function previously exercised by central government has been *devolved* to a non-governmental body, empowered—whether through legislation or some other policy instruments—to conduct that function. As we discuss through three examples below, HRECs can be seen to fit within these broader trends.

By scrutinising HRECs through a regulatory lens, our aim in this article is to locate HRECs within the wider context of devolved governance. Doing so has two useful implications. Firstly, it makes visible that HRECs are acting *as regulators*, and thus allows for a substantive analysis of how well or poorly they are exercising that function. Secondly, understanding HRECs in the context of the regulatory state—rather than as isolated or *sui generis* phenomena—allows us to draw on existing regulatory scholarship, and to apply insights as to how HRECs might be supported to become better regulators. We discuss this below, in Sect. 4.

### Example 1: responsibility for assessing the safety of investigational products

Since 1987, any clinical trial in Australia using a new or modified therapeutic good has been required to satisfy requirements in the federal *Therapeutic Goods Act*. Initially this was managed through the Clinical Trial Approval (CTA) Scheme (previously named the Clinical Trial Exemption (CTX) Scheme),^1^ under which the Australian Therapeutic Goods Administration (TGA) undertakes a direct assessment of product safety. In 1991, the Clinical Trial Notification (CTN) Scheme was introduced as an alternative, more streamlined, review process for lower risk clinical trial proposals (Therapeutic Goods Administration 2018, 17). A key driver in this regard was a 1990 Report of the Australian National Council on AIDS Working Party on the Availability of HIV/AIDS Treatments, which was commissioned to advise on any causes of delay in initiating clinical trials and improving early access to drugs. The review recommended that the CTA scheme be supplemented by a notification scheme “whereby the sponsor company notifies the Therapeutic Goods Administration that a trial is to proceed following approval by the responsible institutional ethics committee” (Australian National Council on AIDS. Working Party on HIV/AIDS Treatments 1991 Rec 5).

As it presently operates, the CTN scheme requires trial sponsors to submit all material relating to the proposed trial to the reviewing HREC. The TGA does not review any data relating to the trial, however, it may ask questions or request additional information should it form “a degree of concern” (Therapeutic Goods Administration 2018, 19) Instead, the HREC is responsible for assessing the trial”s scientific validity, risk–benefit ratio, and overall ethical acceptability (Therapeutic Goods Administration 2018, 19). Paragraph 3.1.4 of the *National Statement* specifies criteria for HREC approval of clinical trials, including:

- The likelihood of therapeutic benefit from the intervention
- Whether there is a “realistic possibility” that the intervention will be at least as beneficial as standard treatment
- Whether any risks of participation are justified by potential benefits to which participants attach significance

HRECs also must review the overall merit of research proposals, including their likely contribution to knowledge and understanding, the appropriateness of the research methods, and its grounding in current literature (National Health and Medical Research Council et al. 2007, para. 1.1(a)).

Despite early suggestions to limit the kinds of trial suitable for the CTN scheme (e.g. to trials already approved by USA or UK authorities) (Australian National Council on AIDS. Working Party on HIV/AIDS Treatments 1991 Rec 7), no formal constraints were initially placed on the use of the CTN scheme (Later on, trials involving “class 4 biologicals” that don’t yet have clinical evidence to support their use were excluded from its scope).^2^ The result has been a burgeoning of the Scheme”s prominence in the Australian clinical trial landscape. The most comprehensive review in this regard was conducted in 2005 by Alan Bansemer, in his *Review of Clinical Trials and Access to Unapproved Therapeutic Goods*. The mandate of the so-called Bansemer Review was, in part, to identify and make recommendations to remove existing barriers to clinical research in Australia (Banscott Health Consulting 2005). The operation of the CTN scheme was a significant focus of the Review. The Bansemer Review noted a significant expansion of CTN as compared with CTA trials in Australia. In 1991, when the scheme was introduced, 42 CTA trials were reported and 63 CTN trials. By 2000, this had shifted to just two CTA trials and 541 CTN trials.

The Bansemer Review concluded that—although the CTN scheme was highly effective in promoting clinical trial activity in Australia—its success placed a corresponding burden on HRECs that necessitated resourcing (Banscott Health Consulting 2005, 11). The Review identified the TGA”s “very strong reluctance” to review clinical trial applications (Banscott Health Consulting 2005, 72), and echoed claims from public interest bodies that the CTN scheme had “greatly reduced the involvement of the TGA and transferred the workload of review of clinical drug trials to HRECs” (Banscott Health Consulting 2005, 67), with the consequence that committees were diverted from carrying out their traditional role of protecting research participants from harm.

In responding to the Bansemer Review, the Australian Government accepted that clinical trial regulation was “significantly institution-based” (Australian Government 2006, 4), and noted questions that had been raised in the past “about whether the CTN Scheme would afford adequate protection for trial participants and whether ethics committees would be able to cope with additional responsibilities and pressures placed on them” (Australian Government 2006, 25). However, relevant changes in the Government response were essentially limited to the development of guidance documents for HRECs on regulatory requirements and the evaluation of trial documentation (Australian Government 2006, 14).

Available data on clinical trials presently being conducted in Australia suggests that the division between the CTN and CTA schemes scheme remains heavily tilted towards CTN. This remains true even in the context of early phase trials: arguably those trials that require the greatest level of specialised scrutiny. A July 2019 Freedom of Information request (FOI Request FOI 1110-1819) revealed that in the five years between July 2013 and July 2018, the TGA assessed only eight clinical trial products under the CTA scheme. Over this same time period, more than 3500 new Australian clinical trials were listed on publicly available registries, over 500 of which were for early phase (phase 0 and 1) trials.^3^

There are clear drivers for the expansion of the CTN as compared with the CTA scheme in Australia. Market-based incentives for use are an important starting point. As at December 2021, submission under the CTN scheme costs sponsors $380, whereas a submission under the CTA scheme can cost from $1810 to $27,400 in the case of higher-risk medicines such as biologicals (Therapeutic Goods Administration 2021). Assessment by HRECs under the CTN scheme also might be faster than the CTA scheme.

Alongside these commercial drivers sits considerable sponsor choice in decisions about whether to seek review for a trial under the CTN or CTA schemes. The only trials for which review under the CTA scheme is mandated are “class 4 biologicals” that don’t yet have clinical evidence to support their use.^4^ The Australian Clinical Trial Handbook provides additional information for sponsors, advising that “The CTA route is generally for high risk or novel treatments, such as gene therapy, where there is no or limited knowledge of safety” (Therapeutic Goods Administration 2018, 17). However, the Handbook also notes that “The CTN scheme may be used for earlier phase studies if there is adequate preclinical information available, especially regarding safety” (Therapeutic Goods Administration 2018, 18). In accordance with prior reviews of the CTN scheme, the Handbook specifies that “A HREC may determine that it does not have access to the appropriate scientific and technical expertise to review the proposed trial under the CTN scheme and recommend review under the CTA scheme” (Therapeutic Goods Administration 2018, 18). In totality, this guidance suggests the availability of the CTN scheme for almost all trials, with the potential for HRECs to refer trials to the CTA scheme if it considers this to be necessary for an adequate review. No data is available on HREC use of this referral pathway, however, the low number of CTA applications overall suggests any such referrals are limited.

### Example 2: authorising the use of personal information for health and medical research under privacy laws

A further responsibility with which HRECs have become entrusted is the acceptability of the collection, use, and disclosure of personal information for the purpose of health and medical research.

Legislative privacy protections were introduced in Australia in 1988 with the enactment of the *Privacy Act 1988* (Cth). Sections 95 and 95A of the Act permits the NHMRC to issue guidelines for the protection of privacy in health and medical research by federal public sector agencies and private sector organisations respectively.^5^ The guidelines require an HREC to make an assessment about the public interest in maintaining privacy protection as compared with the public interest in the proposed research. If the interest in the research activity substantially outweighs the interest in maintaining adherence to the privacy principles under the Act, the HREC may authorise the research. In order to make this assessment, the s 95 Guidelines (those applicable to the federal public sector) require researchers to provide HRECs with information about:

- the aims or purpose of the research
- the credentials and technical competence of the researcher
- the data needed and how it will be analysed
- if sensitive information is to be used, why it is necessary
- the source of the data
- the study period
- the target population
- the reasons why de-identified information cannot achieve the relevant purpose of the research activity
- the reasons why it is impracticable to seek consent from the individual for the use or disclosure of the personal information
- the specific uses or disclosures that will be made of the personal information
- the proposed method of publication of results of the research and a statement that any personal information to be used or disclosed will not be published unless in de-identified form
- the estimated time of retention of the personal information
- the identity of the custodian(s) of the personal information used during the research
- security standards to be applied to the personal information. In particular, that personal information will be retained in accordance with Chapter 2 of the *Australian Code for the Responsible Conduct of Research, 2007*, and in a form that is at least as secure as it was in the sources from which the personal information was obtained unless more stringent legislative or contractual provisions apply
- a list of personnel with access to the personal information including any contractors or subcontractors
- the standards that will be applied to protect personal information disclosed by an agency. These should include the:
  i. terms of any disclosure agreement between the agency and the researcher to govern the limits on use and disclosure of that personal information
  ii. proposed methods of disposal of the personal information on the completion of the research, and that these are in accordance with the Archives Act 1983 for Commonwealth records and relevant legislative requirements of a State or Territory
  iii. standards that will be applied to protect privacy of personal information where it is made available to other researchers or third parties if that is proposed
- any proposal to send data overseas for the purpose of the research project including the names of the countries to which it is proposed the data be sent and how the research project will comply with APP 8 of the Privacy Act (National Health and Medical Research Council 2014b).

Similar disclosure requirements apply under the s 95A guidelines (National Health and Medical Research Council 2014a). In an independent review of the Australian privacy framework, as it relates to health information, Professor Colin Thomson noted that this level of disclosure “significantly exceeds the information that an HREC would normally require from a researcher for the purpose of reviewing and deciding on the ethical acceptability of a research proposal” (Thomson 2004, 7).

The Guidelines further require an HREC, when reviewing this information, to assess whether it “has sufficient information, expertise and understanding of privacy issues, either amongst the members of the HREC or otherwise available to it, to make a decision that takes proper account of privacy” (National Health and Medical Research Council 2014b, para. 3.1). In making its decision, an HREC must consider the privacy principles that might be breached by the research activity, the necessity for the research to use identified or potentially identifiable data, and the reasonableness of the research proceeding without individual consent. HRECs also must satisfy detailed record-keeping obligations (National Health and Medical Research Council 2014b, para. 3.4). In sum, these requirements vastly extend HREC decision making beyond a “peer review” model to something much more akin to a regulatory responsibility. They also suggest the need for HRECs to embark on statutory interpretation: a task for which HREC membership was not originally designed (Thomson 2004, 7–8).

The totality of the above supports a contention that HRECs perform a regulatory role under Australian privacy laws in terms of assessing the acceptability of waiving individual privacy interests to promote societal interests in health and medical research. This is in contrast, for example, to the Confidentiality Advisory Group: a statutory body that operates in England and Wales to advise the Health Research Authority on the acceptability of access to personal information without consent for the purpose of medical research (*Health Service (Control of Patient Information) Regulations 2002 (Eng & Wales)*; *National Health Service Act 2006 (Eng & Wales)*, sec. 251).

### Example 3: authorising research projects where the participants are incapacitated

The third example of regulatory responsibility comes from Australian state and territory law. In Australia, guardianship law is a state and territory matter, and each state and territory has its own legislative regime for the appointment and review of guardians for adults and young people and the regulation of consent to medical treatment for people who are not capable of making healthcare decisions. A feature of these regimes is their classification of types of medical intervention so that “special” medical treatments may require approval by a tribunal, or a more rigorous form of consent by a guardian, before an incapacitated person is permitted to be a patient. The Australian Capital Territory, New South Wales, Victoria and Western Australia all include medical research in this category (*Guardianship Act 1987 (NSW)*, sec. 45AA; *Guardianship and Administration Act 1990 (WA)*, sec. 110ZR; *Guardianship and Administration Act 2000 (Qld)*, sec. 72 See Re application by Dr Matthew Hope [2012] QCAT 191; *Guardianship and Management of Property Act 1991 (ACT)*, sec. 34; *Medical Treatment, Planning and Decision Act 2016 (Vic)*, sec. 75). Additionally, all these jurisdictions (barring the Australian Capital Territory) require the research to be “approved” by a HREC before the tribunal can provide its own approval for the research to go ahead (*Guardianship Act 1987 (NSW)*, sec. 45AA; *Guardianship and Administration Act 1990 (WA)*, sec. 110ZR; *Guardianship and Administration Act 2000 (Qld)*, secs. 72, 74C; *Guardianship and Management of Property Act 1991 (ACT)*, sec. 34; *Medical Treatment, Planning and Decision Act 2016 (Vic)*, sec. 75).

In the Australian Capital Territory, s 34(2)(a) of the *Guardianship and Management of Property Act 1991 (ACT)*, allows for a guardian to consent to a protected person being a participant in medical research but requires the research to be “approved.” The section does not specifically state that the approved needs to by an HREC but we would argue that this is the only probable interpretation. In New South Wales, the consent under s45AA(2)(e) of the *Guardianship Act 1987* (NSW) can be given for an incapacitated person”s involvement in a “clinical trial” but only after that trial has been approved by the Tribunal, with one of the criteria of approval being that “the trial has been approved by a relevant ethics committee and complies with any relevant guidelines issued by the National Health and Medical Research Council.”

In Queensland, the legislation distinguishes between “special medical research or experimental health care” (medical research where the tribunal must give individualised consent for each participant) and “clinical research” (where consent can be given by substitute decision-maker after the project has been approved by the tribunal). In practice, these categories have been hard to distinguish, as was evident in the case of *Re application by Dr Matthew Hope* [2012] QCAT 191. Nevertheless, both categories of treatment may be approved by the tribunal only when the research has been approved by an ethics committee.

In Victoria, substitute decision-makers (“medical treatment decision makers”) may provide consent to an incapacitated person”s participation in research, but the research must have been approved by an HREC (*Medical Treatment, Planning and Decision Act 2016 (Vic)*, sec. 75(a)). Emergency research can also be performed on incapacitated persons without the consent of a medical treatment decision maker, with one of the conditions being that the research has been approved by an HREC, where the HREC knew that the research may be conducted without the consent of the person or their medical treatment decision maker (*Medical Treatment, Planning and Decision Act 2016 (Vic)*, sec. 80(1)(b)). It is a legislative requirement that any research be carried out according to the terms of the HREC approval (*Medical Treatment, Planning and Decision Act 2016 (Vic)*, sec. 76).

Finally, in Western Australia, substitute decision-makers may consent to an incapacitated person being a participant in medical research, but this is, once again, conditional on the research being approved by an HREC (*Guardianship and Administration Act 1990 (WA)*, sec. 110ZR(1)(a)). Research in emergency situations is also permitted under s 110ZS(1)(a) of the *Guardianship and Administration Act 1990* (WA), based on the condition of prior HREC approval.

These examples, evidence that HRECs are an important part of the regulatory process of medical research involving incapacitated patients at the state and territory level, in addition to the federal functions they perform that were described above.

## “Regulatory scaffolding” for HRECs?

Section 3 evidenced that, despite being originally intended as peer review bodies, HRECs in Australia are today exercising a regulatory role. Although the case studies provided are Australian, it is likely that this same transition has occurred in other countries, such as the United States. A number of US scholars have described IRBs as a “regulatory system”, commonly in combination with criticism of the system”s operation (Burris 2008; Heimer and Petty 2010), such as Scott Burris”s lament that “though ill-equipped for the task, the IRB is now an oversight agency expected to spot bad actors and monitor researcher behavior” (Burris 2008). Holly Fernandez Lynch has described the IRB role as “regulatory gatekeepers” whose “existence is mandated by regulation” and role is “overseeing research according to regulatory terms” (Lynch 2018, 151).

The regulatory role of HRECs and their international equivalents is potentially problematic not only on a substantive level (in terms of risks to participants), but also on a procedural one (Committees were never intended, nor designed, to be regulators). Explicit recognition of the regulatory role of HRECs also evidences some of the limitations of prior recommendations for reform. As synthesised by Scott Burris,

Bioethicists and a succession of expert investigations have identified the regulatory defects of the IRB, but defined the remedy as more of the same—more review, more documentation, more training (Burris 2008).

Similar remedies have been proposed in the Australian context, in which reports and consultancies have focused on the provision of institutional resources for HRECs (Australian Law Reform Commission 2003 Rec 17-3); requirements for additional reporting by HRECs to government regulators (Australian Law Reform Commission 2003 Rec 15-1); the implementation of HREC quality assurance and accreditation programs (Australian Law Reform Commission 2003 Rec 17-1 and 17-2); and the provision of additional training, support, and advice to HRECs by Government bodies (Australian Law Reform Commission 2003 Rec 15-3; Banscott Health Consulting 2005 R 14, 15 & 21).

While the suggestions above are helpful, they essentially accept the “peer review” model of HRECs and IRBs. They largely fail to challenge or critique the regulatory role that has been bestowed on HRECs, including their role in outsourced public service delivery. We contend that this failure stems from not engaging with HREC review *as* regulation. In this section, we draw on a framework from regulatory studies, specifically Reeve and Magnusson’s model of “regulatory scaffolding” to make some pragmatic and incremental suggestions for reform.

### What does regulatory scaffolding involve?

The discipline of regulatory scholarship has long been engaged with the challenge of achieving “better regulation”—that is, improving regulatory processes, and better achieving the aims of regulation. Reeve and Magnusson advanced this tradition in their series of articles on “regulatory scaffolding” in the context of chronic disease prevention, specifically relating to self-regulation by the food industry (Reeve and Magnusson 2013, 2015; Magnusson and Reeve 2014). Food industry self-regulation is a classic example of devolved governance using “soft” regulatory options. In this case, the industry undertakes to regulate to the nutritional quality of its products on a voluntary basis, using self-developed codes or guidelines, often in an effort to fend off “harder” government oversight. This approach has received significant criticism – most seriously, because it has not been effective. However, public health observers acknowledge that these soft regulatory options are currently the only feasible ones. This being the case, Reeve and Magnusson approach the problem pragmatically. Drawing on regulatory scholarship, they propose that, in circumstances where command-and-control regulation is lacking or not feasible, soft regulatory instruments and devolved structures might nevertheless be “scaffolded” to improve their effectiveness. They propose an approach of progressively increasing the level of government oversight or control over industry players in response to industry inaction or non-compliance with existing regulations. This approach acknowledges current political realities, while still seeking to improve the status quo of regulation. As Reeve and Magnusson write, in circumstances where “prescriptive technical controls… remain out of reach”, governments nevertheless need to be able to “go beyond education and to consider novel forms of action to improve public health” (Reeve and Magnusson 2013, 495).

The concept of regulatory scaffolding is underpinned by two basic propositions. The first is that, even where they choose to devolve some regulatory responsibility to a non-governmental body, governments nevertheless retain the duty to safeguard the public interest. As Magnusson and Reeve note, “where governments permit private institutions to self-regulate, they have a responsibility to act as guardians of the public interest, by ensuring that regulatory processes do not prioritise private interests at the expense of legitimate public health goals” (Magnusson and Reeve 2014, 273). The second proposition comes from regulatory scholarship: essentially, that governments have a wide range of regulatory options at their disposal, beyond mandatory standards. Magnusson and Reeve write:

In circumstances when it is not prescribing regulatory standards, governments may nevertheless: set goals and indicators of success; structure the relationships between different groups that are directly involved in regulation; ensure that regulatory processes are transparent and accountable; and evaluate the extent to which private forms of regulation are successful in achieving public goals (Magnusson and Reeve 2014, 274).

On that basis, what does regulatory scaffolding entail? Drawing on the concept of responsive regulation, Reeve and Magnusson propose “the introduction of regulatory and legislative “scaffolds” to strengthen the performance and credibility” of existing structures (Reeve and Magnusson 2013, 495). Such scaffolds might include:

1. Setting clear goals – including defining the respective roles and responsibilities of key parties – and measurable targets and indicators for measuring success or failure.
2. Closing off significant escape clauses in self-regulatory regimes.
3. Holding industry to account for its voluntary commitments. Governments can enhance the transparency and accountability of self-regulation through official monitoring and public disclosure of industry performance, and by requiring registration and independent administration of voluntary codes, and finally,
4. The existence of a “credible expectation that government will escalate levels of intervention” where the devolved or soft regulatory strategies are failing to achieve their intended aims (Reeve and Magnusson 2013, 496).

### What might regulatory scaffolding look like for HRECs?

While there are clear differences between regulating innovative medical research, and regulating the industrial vectors of chronic disease, there are also important parallels which suggest that regulatory scaffolding could be of value in relation to the quality of regulation provided by HRECs. The first of these is an overarching governmental responsibility to safeguard public health—including the health and wellbeing of research participants, and the second is a lack of existing effective command-and-control regulation. Drawing on Reeve and Magnusson’s work as summarised above, we make the following proposals for how regulation by HRECs might productively be “scaffolded”.

#### Setting clear roles and responsibilities, along with targets for measuring success or failure

There is a proliferation of guidance documents for the ethical acceptability of human research; however, limited information on what is expected of review Committees or measures for effectiveness (Abbott and Grady 2011). To the extent that goalposts have been set, they typically relate to procedural as compared with substantive metrics. For example, the Australian *National Certification Scheme of Institutional Processes Related to the Ethical Review of Multi-centre Research* requires HRECs seeking certification to demonstrate the availability of terms of reference and standard operating procedures, provision of annual reports, and so forth (National Health and Medical Research Council 2020). However, it does not assess applications that have been approved by the Committee or the stipulations the Committee may have imposed on such applications. Roles and responsibilities also could be helpful to delineate the accepted scope of the CTN Scheme, along with a clear obligation on HRECs to decline to review applications in certain circumstances: for example, if it is unable to access the expertise necessary to review product safety for an application submitted under the CTN Scheme. Of course, setting higher standards for reporting does not of itself improve the substantive quality of the decisions being made. It will, however, give the Australian public a better understanding the work that is actually being done.

#### Holding HRECs to account for their decision making

Arguably there is a need for much more accountability about HREC decision-making. By “accountability” we mean the value of decision-makers being required to provide justifications for their decisions, and to change their behaviour when they have been found to have breached norms of conduct (Nielsen et al. 2021). The benefit of making HRECs accountable is that it would help to raise the consciousness of HREC members, encouraging them to reflect on whether their decisions accord with the norms under which they operating.

Equally important is transparency. If HRECs are to be made accountable for decisions, that can only occur if there is a transparency about how their decisions have been made. Presently information is only usually available through freedom-of-information processes, and sometimes refused under this scheme, as was the case in *Battin v University of New England* [2013] NSWADT 73 (See also Lynch 2018).

A realistic mechanism of accountability and transparency would be a requirement for HRECs to provide reasons for their decisions that could be deposited in a public database. A library of prior decision-making should also be available from which to draw precedents. Having such a library would encourage consistency and improvement in the quality of decision-making (Seykora et al. 2021). Furthermore, HRECs should be required to justify their expertise to review CTN trials and should be audited on such. Any concerns regarding commercially sensitive information could be dealt with by appropriate protocols for de-identification as are employed in other published decisions.

#### Holding clinical trial sponsors to account for their use of the HREC system

In addition to increasing the levels of transparency and accountability of HRECs, we believe that clinical trial sponsors should be required to justify their choice of CTN or CTA schemes. Market-based forces can”t be enough to propel a trial through CTN that raises novel scientific issues requiring specialist review, including first-in-human trials of novel drugs and devices. Wide-ranging evidence that early-phase trials are being propelled through CTN should trigger escalation, as described below.

#### Credible expectation of escalation and review

Accountability also requires the credible existence of regulatory review. That review could take many forms. Firstly, it could the take the form of some kind of quality assurance, where HRECS would have to audit their own behaviour to analyse how well they are meeting regulatory review standards (especially product safety, privacy protections; consent of incapacitated adults). HRECs could also monitor the prevalence of CTA and CTN applications to see how well industry is demarcating between the two, and whether sponsors are using CTA for trials for which HREC expertise is unlikely to be adequate. Crucially, should these reviews show ongoing problems, there must be an expectation that some form of reporting be made to the NHRMC, such as already exists in compliance reporting (e.g., number of meetings held, membership categories filled, etc.), to enable the NHMRC to investigate and provide avenues for redress. All of this could be achieved by expanding on the current requirements for HRECs to perform compliance reporting.

A further, and as yet untapped, mechanism of review would be to make HRECs subject to administrative law or judicial review of administrative action (McWhirter 2022). This would mean that HREC decisions would be subject to review by the either the Federal or State administrative decisions tribunals, on the grounds that decisions were, broadly, beyond power, procedurally unfair, or irrational.

Traditionally, HRECs have not been understood as being subject to judicial review, perhaps on the grounds that they were not performing governmental functions, but this is a hard position to maintain given the way that HRECs are embedded into regulation. For example, the Australian Law Reform Commission recognised the potential for HRECs to be subject to judicial review when making decisions under the ss 95/95A Guidelines issued under the Privacy Act, discussed above.

#### The economics of HRECs

Finally, the economics of these proposals also needs to be considered. There is no doubt that extra compliance and accountability mechanisms will lead to increase economic burdens on HRECs. But we would argue that any economic analysis should also account for the improvements in the quality of decision-making.

## Conclusion

In this paper, we have sought to understand the evolution of the role of HRECs by understanding these bodies as regulators, operating within a wider regulatory context. We began by outlining the history of HRECs and the growth in their scope of function. We then situated HRECs within the regulatory studies literature as an example of a “devolved regulator” and we illustrated that claim against three areas of HREC activity in Australia – testing of the safety of investigational products; the consideration of release of personal data without consent; and the approach of research involving mentally incapacitated participants. We believe that these areas display the role of HRECs as regulator, as they play key decision-making role in the administration regulatory power and authority.

We then applied a regulatory scaffolding approach to HRECs drawing on the work of Reeve and Magnusson and we made five proposals for improving the regulatory functionality of HRECs. As with IRBs in the US, HRECs play an absolutely pivotal role in the research enterprise in Australia and it is essential that they operate well. Understanding HRECs as regulators gives us a more accurate picture of the functions of HRECs, what we want them to achieve, and how we can improve their performance.

## Data Availability

Available from authors on request.

## Footnotes

1 The CTX Scheme was renamed the CTA scheme on 6 November 2020 to more accurately reflect the nature of scheme: https://www.tga.gov.au/clinical-trial-exemption-ctx-scheme-renamed-clinical-trial-approval-cta-scheme. The scheme”s operation remained unchanged. For ease of readability, the scheme will be referred to as CTA throughout this article, even though much of the data was collected under the name of CTX.

2 “Class 4 biologicals” are defined in s 1 of sch 16 of the *Therapeutic Goods Regulations 1990* (Cth). The definition includes: “(a) biologicals that comprise or contain: (i) live animal cells; or (ii) live animal tissues; or (iii) live animal organs; (b) biologicals to which both of the following paragraphs apply: (i) the biologicals comprise, contain or are derived from human cells or human tissues that have been modified to artificially introduce a function or functions of the cells or tissues; (ii) the artificially introduced function or functions were not intrinsic to the cells or tissues when they were collected from the donor; (c) pluripotent stem cells; (d) biologicals derived from pluripotent stem cells”.

3 Based on a search of www.anzctr.org.au for trials recruiting in Australia listed between 1/7/2013 and 1/7/2018 that had been approved by an ethics committee and listed on either ANZCTR or ClinicalTrials.Gov. Selected trial codes were Treatment: Drugs and Treatment: Devices, excluding other interventions such as Treatment: Surgery and Prevention (which might not require TGA approval).

4 “Class 4 biologicals” are defined in s 1 of sch 16 of the *Therapeutic Goods Regulations 1990* (Cth). The definition includes: “(a) biologicals that comprise or contain: (i) live animal cells; or (ii) live animal tissues; or (iii) live animal organs; (b) biologicals to which both of the following paragraphs apply: (i) the biologicals comprise, contain or are derived from human cells or human tissues that have been modified to artificially introduce a function or functions of the cells or tissues; (ii) the artificially introduced function or functions were not intrinsic to the cells or tissues when they were collected from the donor; (c) pluripotent stem cells; (d) biologicals derived from pluripotent stem cells”.

5 Similar provisions exist at the state level, eg, See *Privacy and Personal Information Protection Act 1998* (NSW), s 27B; *Health Records and Information Privacy Act 1998* (NSW), s 27B, and *Statutory Guidelines on Research – Sect. 27B* (2019) https://www.ipc.nsw.gov.au/sites/default/files/2020-01/Statutory_Guidelines_on_Research_section_27B_September_2019.pdf and *Statutory Guidelines on Research* (2004) https://www.ipc.nsw.gov.au/sites/default/files/file_manager/privacy_statutory_guidelines_research.pdf.
